# Outcomes of subretinal macular hemorrhage treatment: a 7-year retrospective cohort study at Oslo University Hospital

**DOI:** 10.1186/s40942-025-00749-3

**Published:** 2025-11-22

**Authors:** Krystian Andrzej Dziedzic, Filippo Confalonieri, Beáta Éva Petrovski, Lyubomyr Lytvynchuk, Ragnheidur Bragadottir, Ivan Borjan, Xhevat Lumi, Goran Petrovski

**Affiliations:** 1 Center for Eye Research and Innovative Diagnostics, Department of Ophthalmology, Oslo University Hospital and University of Oslo, Oslo, 0424 Norway; 2 Department of Biomedical Sciences, Humanitas University, Pieve Emanuele, 20090 Milan, Italy; 3 Department of Ophthamology, IRCCS Humanitas Research Hospital, Rozzano, 20089 Milan, Italy; 4 Department of Ophthalmology, Justus Liebig University Giessen, University Hospital Giessen and Marburg GmbH, Giessen, Germany; 5 Department of Ophthalmology, Karl Landsteiner Institute for Retinal Research and Imaging, Vienna, Austria; 6 Department of Ophthalmology, University Hospital Split, Spinciceva 1, Split, 21000 Croatia; 7 School of Medicine, University of Split, Soltanska 2, Split, 21000 Croatia; 8 UKLONetwork, University St. Kliment Ohridski-Bitola, Bitola, 7000 North Macedonia

**Keywords:** Subretinal macular hemorrhage (SRMH), Anti-VEGF therapy, Tissue plasminogen activator (tPA), Pars plana vitrectomy (PPV), Best corrected visual acuity (BCVA), tPA and gas displacement, Pneumatic displacement

## Abstract

**Purpose:**

Subretinal macular hemorrhage (SRMH) is a vision-threatening condition with no consensus on optimal treatment. This study evaluated the outcomes of five treatment modalities and their correlation with patient characteristics and optical coherence tomography findings.

**Methods:**

A retrospective review was conducted on 120 SRMH patients treated at Oslo University Hospital between October 24, 2015, and December 27, 2022. Best-corrected visual acuity (BCVA) was assessed before and 1-month after treatment. Statistical analyses were performed to evaluate the impact of treatment timing, hemorrhage size, and different therapeutic interventions on visual outcomes.

**Results:**

Early intervention, particularly within 7 days of symptom onset, was associated with better median BCVA 1-month post-surgery. A 14-day cut-off showed no benefit. Patients with smaller hemorrhages (< 4500 μm) had significantly higher BCVA before treatment, but no statistical difference was observed 1-month after treatment. Combination therapies, including tissue plasminogen activator (tPA) with Sulfur Hexafluoride (SF6) gas and tPA with SF6 gas plus anti-vascular endothelial growth factor (anti-VEGF), resulted in significantly improved visual outcomes 1-month post-surgery, but no intervention outperformed others at the 1-month endpoint.

**Conclusions:**

Early intervention, particularly within 7 days of symptom onset, may improve visual outcomes in SRMH. Combined therapies involving tPA, gas and anti-VEGF appear to be more effective. Further studies with extended follow-up are needed to establish definitive treatment guidelines.

**Translational relevance:**

This study highlights the importance of early intervention and individualized treatment strategies in managing SRMH. The findings contribute valuable insights into the effectiveness of combination therapies, offering guidance for clinicians and informing future research on evidence-based treatment guidelines.

**Supplementary Information:**

The online version contains supplementary material available at 10.1186/s40942-025-00749-3.

## Introduction

Subretinal macular hemorrhage (SRMH) causes an immediate loss of central vision, and is of major concern due to its capacity to inflict irreversible severe damage if left untreated [1]. The condition is characterized by an accumulation of blood between the neurosensory retina and the retinal pigment epithelium (RPE) [2], and can be linked to various etiological factors. These include neovascular age-related macular degeneration (nAMD), polypoidal choroidal vasculopathy, retinal arterial macroaneurysms, blunt trauma and other causes [3–7]. The complexity of SRMH, coupled with its clinical significance, has spawned an ongoing debate regarding the optimal treatment strategy [8, 9], primarily due to an insufficient number of patients in prospective studies and a consequent lack of a universally accepted best practice. Despite the advancements in diagnostic techniques and therapeutic interventions, the management of SRMH remains complex, and varies widely in approach and efficacy.

The incidence of SRMH is uncertain as small hemorrhages are usually not treated surgically, but lesions larger than 2 disc diameter affect approximately 25 people per million annually according to one study [10]. The anticipated increase in prevalence due to demographic shifts towards an aging population is particularly concerning given the condition’s severe implications for vision, which can range from a visual acuity of 6/30 to mere light perception if left untreated [1].

The pathogenesis of SRMH involves a cascade of biochemical and mechanical processes that compromise the integrity of retinal structure and function. These processes include iron-mediated toxicity, impairment of diffusion of oxygen, and mechanical damage due to clot contraction [11]. All of the above collectively disrupt the metabolic exchange between the retina and its underlying support layers, culminating in subretinal fibrosis, macular atrophy and photoreceptor death [1, 12–15].

A plethora of treatment approaches have been tried over the years, ranging from intravitreal anti-vascular endothelial growth factor (anti-VEGF) injections, to more invasive procedures such as subretinal or intravitreal tissue plasminogen activator (tPA) injections with gas, either alone or combined with anti-VEGF. Anti-VEGF treatment has been shown to be effective in resolving SRMH and decreasing the likelihood of its recurrence by inhibiting the activity of VEGF, a protein promoting the growth of abnormal blood vessels [16, 17]. Pneumatic displacement of SRMH is expected to reduce the risk of iron toxicity exposure and detachment of the neuroretina from the RPE, which in turn mitigates retinal damage [18, 19]. The use of intravitreal or subretinal tPA may assist in dissolving the clot [20], and facilitate pneumatic displacement.

This study aimed to enhance the current understanding of SRMH-treatment by conducting a retrospective review of outcomes from 120 patients treated at Oslo University Hospital (OUH) between 24.10.2015 and 27.12.2022. The effectiveness of the five treatment modalities employed in recent years were assessed using best corrected visual acuity (BCVA) and correlated with structural analysis using optical coherence tomography (OCT). This information was used to identify predictors of enhanced visual outcomes. Additionally, the study aimed to provide insights that could inform future efforts to develop evidence-based guidelines for SRMH management, addressing the significant variability in treatment approaches and outcomes currently observed in clinical practice.

## Methods

This retrospective cohort study was conducted at OUH to evaluate the effectiveness of five treatment modalities for SRMH. Human Ethics and Consent to Participate declarations were obtained from the Internal Review Board (IRB)/Privacy Officer at OUH under the reference number 23/12,472. As the study was retrospective, the clinical trial number was not applicable.

The study conducted a retrospective review of medical records of patients treated at OUH between 24.10.2015 and 27.12.2022. Eligible participants underwent ocular examinations, including BCVA according to logMAR chart, slit lamp biomicroscopy, Optos California (Optos, Inc., Marlborough, MA, USA) retinal imaging system, and spectral-domain OCT using the RS-3000 device (Nidek Co., Ltd., Gamagori, Aichi, Japan). For patients with a BCVA below 0.1, numerical visual acuity was assessed using finger counting and hand motion, with 0.02 subtracted from 0.1 for each meter the patient could count fingers closer than 5 m. The hemorrhage size and localization were quantified using wide-angle fundus photography and OCT at the initial consultation. Collected data included the interval from symptom onset to treatment, patient age at the time of treatment, and BCVA before and after hemorrhage. BCVA from before the SRMH episode was available due to previous treatment for other conditions at OUH. Other collected data included BCVA 1-month after treatment, pre-surgical intravitreal anti-VEGF administration, lens status, comorbidities (both ocular and systemic), and OCT-based analysis of hemorrhage dimensions, including central foveal thickness, height, and horizontal diameter of the subretinal hemorrhage, as well as the affected retinal layers.

Given the real-world design of this study, treatment was personalized based on the operating surgeon’s assessment; tPA (Actilyse) dose subretinally was given as 50 µg in a volume of up to 0.5mL BSS, and intravitreally as 20 µg in 0.1mL BSS. In the former, the eye was filled with 20% Sulfur Hexafluoride (SF6) gas after liquid-air-exchange, and in the later, 0.3mL of pure SF6 was injected; the choice of anti-VEGF agent was bevacizumab at 1.25 mg/0.05mL concentration. All decisions remained within standard institutional ranges. Patients received one of the following interventions: intravitreal anti-VEGF monotherapy, intravitreal tPA with SF6 gas displacement, intravitreal tPA with SF6 gas displacement combined with anti-VEGF, subretinal tPA with SF6 gas displacement, or subretinal tPA with SF6 gas displacement combined with anti-VEGF.

### Statistical analysis

Descriptive statistical analysis was performed; the results are presented in numbers (N) percentages (%), median with interquartile ranges (IQR: Q3-Q1: 25th and 75th percentile) and range (difference between the highest and lowest values). The normality of continuous variables was tested on a histogram, Q-Q plot, and using the Shapiro–Wilks – and/or Kolmogorov–Smirnov tests. Due to the non-normal distribution of the continuous variables, Wilcoxon Signed Ranks test was used to analyse the median differences in case of best corrected visual acuity (BCVA/logMAR) between two dependent groups like between two time points (pre- hemorrhage, post-hemorrhage, day 1 post-surgery, 1-month post surgery). While Mann-Whitney U test was used to detect the median BCVA differences between independent groups: <14 days, ≥ 14 days, < 7 days, ≥ 7 days, max diameter on Optos Retinal Imaging System (< 4500 μm, ≥ 4500 μm) and lens status (phakic/pseudophakic).

Kruskal-Wallis analysis was used to analyse the BCVA median differences between more than two independent groups like surgery types (anti-VEGF, intravitreal tPA + SF6, intravitreal tPA + SF6 + anti-VEGF, subretinal tPA/vitrectomy + SF6; subretinal tPA/vitrectomy + SF6 + anti-VEGF), location (central subfoveal, parafoveal involving umbo, parafoveal not involving umbo), involved retinal layers (under neurosensory retina, combined), antiplatelets and/or anticoagulants (antiplatelets, anticoagulants, both - antiplatelets + anticoagulants, none).

While for pairwise comparisons the Dunn`s nonparametric post hoc test with Bonferroni correction was used. The data was presented on boxplots. All differences were considered significant at *P* < 0.05.

A post-hoc power analysis was performed to assess the adequacy of the sample size. Given the uneven distribution across treatment groups (range: 4–75 patients), the study was primarily powered to detect moderate-to-large differences in BCVA between major subgroups such as treatment timing (< 7 days vs. ≥7 days; <14 days vs. ≥ 14 days) and (< 4500 μm vs. ≥ 4500 μm). With the sample size (e.g., *N* = 10 vs. 120) the cohort provided approximately 80% power to detect medium effect size (Cohen`s d ≈ 0.6–0.7, equivalent to BCVA differences of ~ 0.08–0.10 logMAR) at a two-sided α of 0.05.

In case of missing values, when the distribution of the data was normal and missing values were random, mean imputation was used. Statistical package for STATA (Stata version 17.0; College Station, TX, USA) was used for the statistical analyses.

## Results

Table 1 shows the demographic and clinical characteristics of the 120 study subjects. The median age was 81.9 years (range: 33.6–95.7). The study population included 54 males (45.0%) and 66 females (55.0%). Most participants underwent tPA + gas surgery (N: 75, 62.5%), followed by tPA + gas + anti-VEGF (N: 24, 20.0%), tPA-subretinal-gas (N: 11, 9.2%), anti-VEGF (N: 6, 5.0%) and tPA-subretinal-gas + anti-VEGF (N: 4, 3.3%). Lens status was almost evenly split between phakic (N: 58, 48.3%) and pseudophakic (N: 62, 51.7%) eyes. Most of the subjects did not use antiplatelets and/or anticoagulants (N: 76, 63.9%), while 43 (36.1%) did. The location of the hemorrhages was primarily central sub-foveal (N: 75, 65.8%). The involved retinal layers were mostly combined (N: 82, 75.9%), with fewer cases under neurosensory retina (N: 22, 20.4%).

**Table 1:** Demographic and clinical characteristics of the study population

**Demographic characteristics**	
Study population	120
Gender (male/female)	54/66 (45%/55%)
Age (median, range)	81.9 (33.6–95.7)
**Type of treatment**	
Anti-VEGF	6 (5.0%)
tPA + gas	75 (62.5%)
tPA + gas + antiVEGF	24 (20.0%)
Vitrectomy + Subretinal tPA + gas	11 (9.2%)
Vitrectomy + Subretinal tPA + gas + antiVEGF	4 (3.3%)
**Lens status**	
Phakic	58 (48.3%)
Pseudophakic	62 (51.7%)
**Antiplatelets and/or anticoagulants**	
Yes	43 (36.1%)
No	76 (63.9%)
**Location** ^**1**^	
Central subfoveal	75 (65.8%)
Parafoveal involving umbo	39 (34.2%)
Parafoveal not involving umbo	-
**Involved retinal layers** ^**2**^	
Under neurosensory retina	22 (20.4%)
Combined	82 (75.9%)

tPA: tissue plasminogen activator; VEGF: vascular endothelial growth factor

^1^6 missing values; ^2^12 missing values

Figure 1A illustrates the BCVA at all timepoints grouped by the time of treatment (< 14 days and ≥ 14 days from symptom onset). There were 93 patients in the < 14 days group and 27 in the ≥ 14 days group. For surgeries performed within 14 days of onset, the median BCVA (logMAR) 1-month post-surgery was 1.00 (IQR: 0.60-2.00). In contrast, surgeries performed ≥ 14 days from onset had a lower median BCVA (logMAR) of 0.77 (IQR: 0.70–1.30) at 1-month post-surgery. BCVA medians differed significantly across all timepoints for both groups (< 14 days, *P* < 0.05; ≥14 days, *P* < 0.001), except between day 1 - and 1-month post-surgery(*P* = 1.00). No statistically significant differences could be detected between the groups at the other timepoints.

**Fig. 1A:**
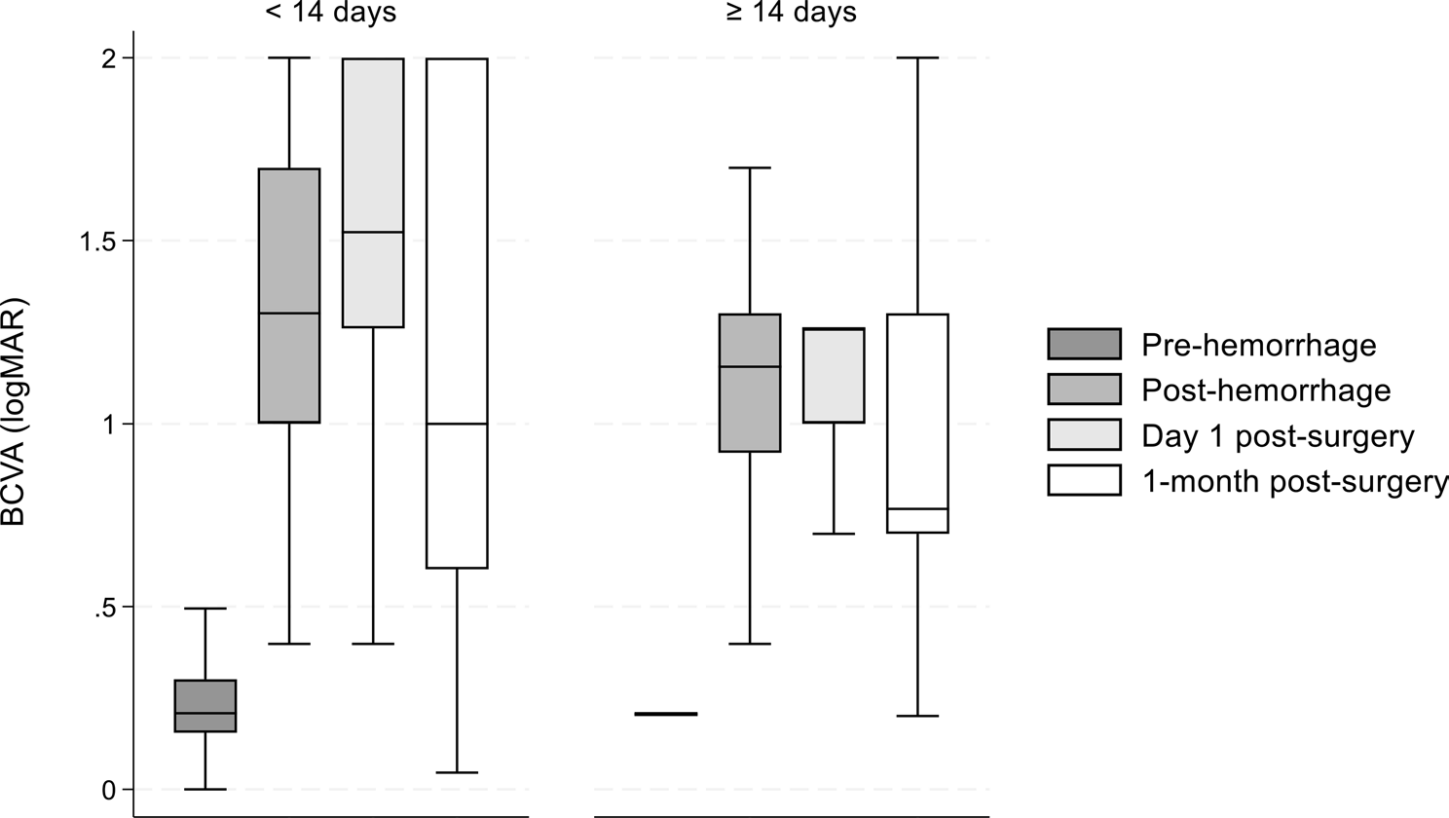
Median BCVA by treatment onset (< 14 days vs. ≥14 days). BCVA: Best corrected visual acuity

Figure 1B presents the BCVA at all timepoints categorized by the time of treatment initiation (< 7 days and ≥ 7 days from symptom onset). There were 10 patients in the < 7 days group and 110 in the ≥ 7 days group. For surgeries conducted within 7 days of onset the median BCVA (logMAR) 1-month post-surgery was 0.65 (IQR: 0.49–1.30). For surgeries performed ≥ 7 days from onset, the median BCVA (logMAR) was 1.00 (IQR: 0.70–1.70) at 1-month post-surgery. Statistically significant differences could be detected in BCVA medians across all timepoints for the < 7 days group (*P* < 0.01), except between post-hemorrhage and day 1-post-surgery, and post-hemorrhage and 1-month post-surgery (*P* = 0.73 and *P* = 0.12). For the ≥ 7 days group, significant differences were observed at all timepoints (*P* < 0.01). Significant differences could be seen in pre-hemorrhage BCVA medians between < 7 and ≥ 7 days (*P* = 0.03).

**Fig. 1B:**
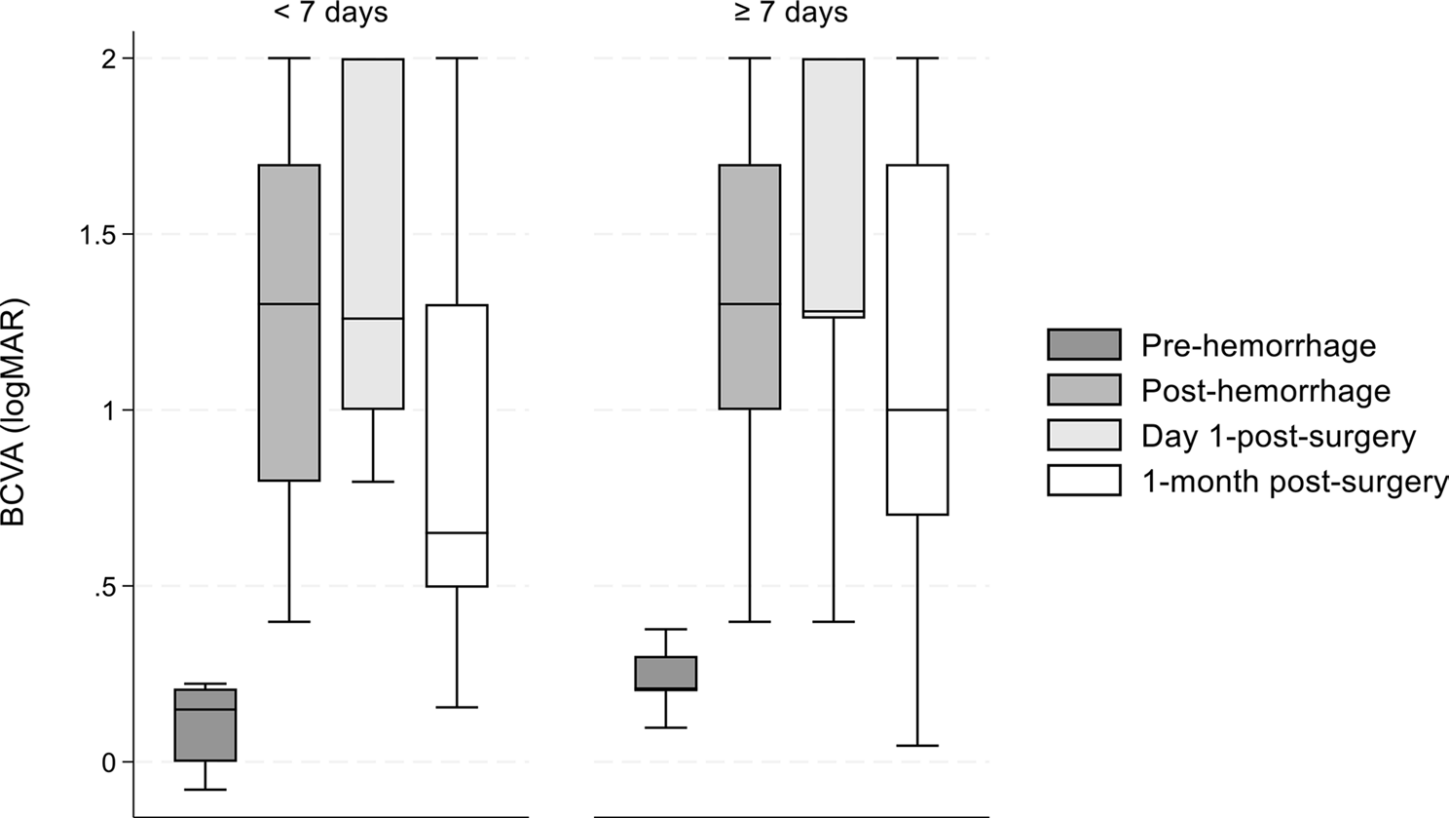
Median BCVA by treatment onset (< 7 days vs. ≥ 7 days). BCVA: Best corrected visual acuity

**Fig. 2:**
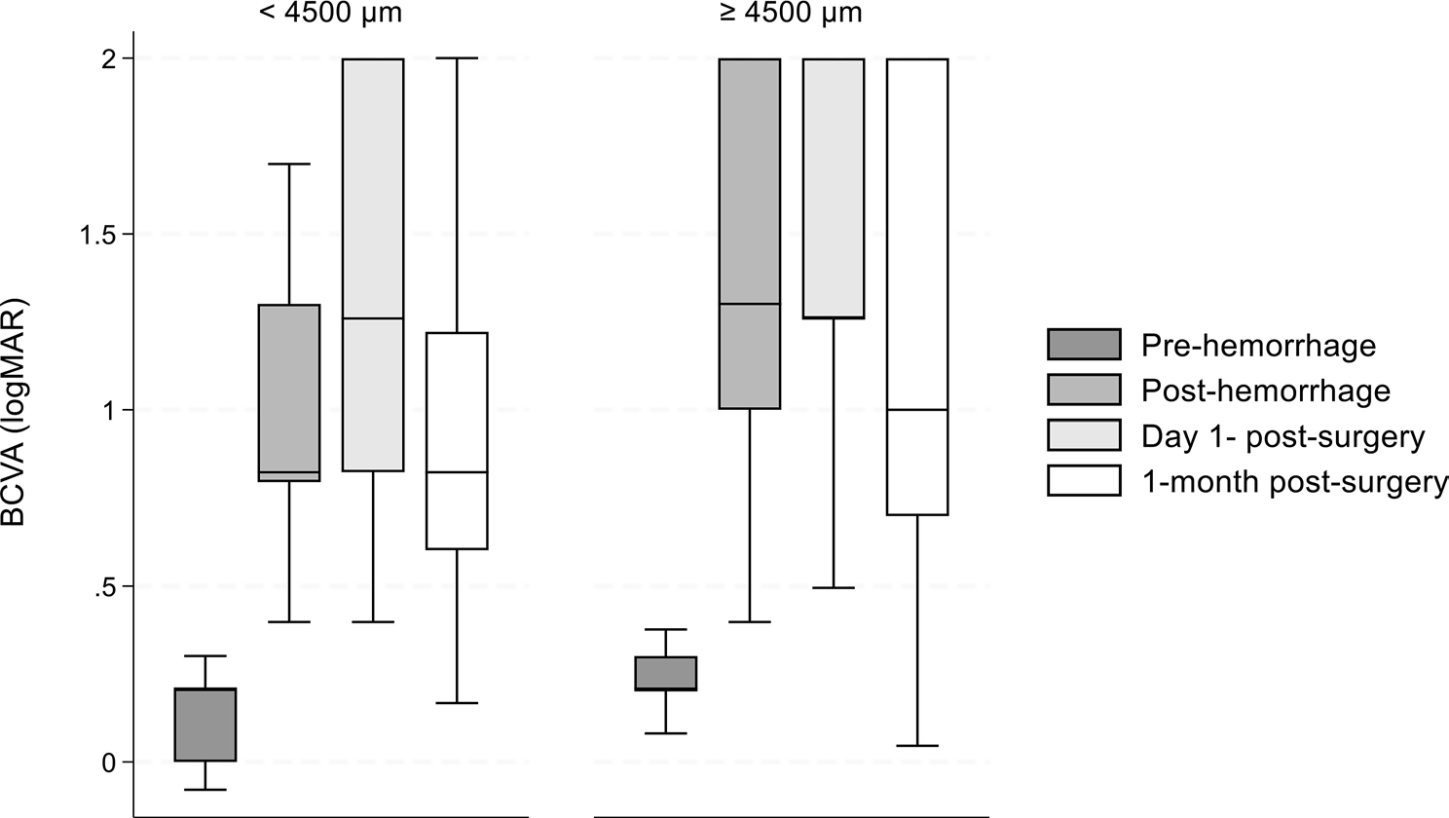
Median BCVA by hemorrhage diameter. BCVA: Best corrected visual acuity

Figure 2 illustrates the BCVA at all timepoints categorized by the maximum diameter of the SRMH. There were 19 patients in the < 4500 μm group and 90 in the ≥ 4500 μm group. For hemorrhages smaller than 4500 μm, the median BCVA (logMAR) 1-month post-surgery was 0.87 (IQR: 0.65–1.23). In contrast, for larger hemorrhages (≥ 4500 μm), the median BCVA (logMAR) 1-month post-surgery was 1.05 (IQR: 0.75-2.00). BCVA medians differed significantly across all timepoints for both groups (< 4500 μm and ≥ 4500 μm, *P* < 0.001), except (< 4500 μm) between post-hemorrhage and day 1 – and post-hemorrhage and 1-month post-surgery (*P* = 0.8), moreover between day 1 and 1-month post-surgery. In case the diameter was ≥ 4500 μm significant median differences could be detected between day 1 - and 1-month post-surgery. A statistically significant difference could be detected only in case of post-hemorrhage BCVA medians between the two diameter groups (*P* = 0.002).

**Fig. 3:**
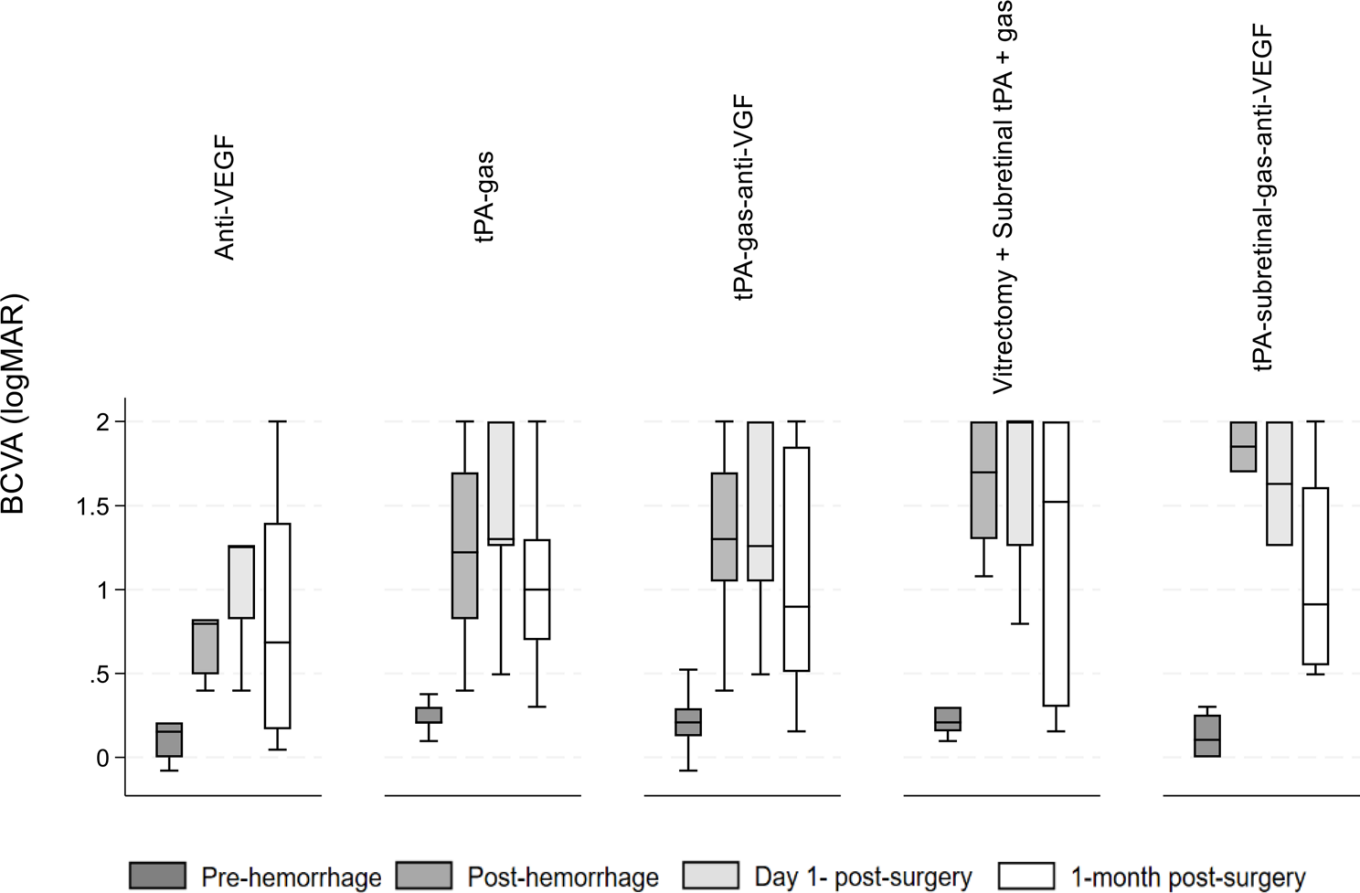
Median BCVA by type of surgery. BCVA: Best corrected visual acuity

Figure 3 shows the median BCVA differences at all timepoints across different surgical interventions. There were 6 patients in the Anti-VEGF group, 75 in the tPA + gas group, 24 in the tPA + gas + anti-VEGF group, 11 in the Subretinal tPA + gas group, and 4 in the Subretinal tPA + gas + anti-VEGF group. After symptom onset, the lowest median BCVA (logMAR) could be observed in case of all types of surgeries at 1-month post-surgery, especially in the anti-VEGF group with a median of 0.68 (IQR: 0.17–1.39), followed by the tPA + gas + anti-VEGF group with a median of 0.90 (IQR: 0.51–1.85). The highest median BCVA (logMAR) could be observed at post-hemorrhage in the tPA + subretinal + gas + anti-VEGF group with the median of 1.85 (IQR: 1.70-2.00).

BCVA medians differed significantly across all timepoints for the Anti-VEGF group (*P* < 0.05), except between post-hemorrhage and day 1 post-surgery (*P* = 0.4), post-hemorrhage and 1 month post-surgery (*P* = 0.9), and day 1 post-surgery and 1 month post-surgery (*P* = 0.6). For the tPA-gas group, BCVA medians differed significantly at all timepoints (*P* < 0.05). In the tPA + SF6 + anti-VEGF group, significant differences were observed in BCVA medians across all timepoints (*P* < 0.001), except between post-hemorrhage and day 1 post-surgery (*P* = 0.5). For the subretinal tPA + SF6 group, significant differences were noted across all timepoints (*P* < 0,01), except between post-hemorrhage and day 1 post-surgery (*P* = 0.9), post-hemorrhage and 1 month post-surgery, and day 1 post-surgery and 1 month post-surgery (*P* = 0.046). The subretinal tPA + SF6 + anti-VEGF group did not show significant differences across timepoints.

BCVA medians differed significantly between the different surgery types for post-hemorrhage (*P* < 0.0049). The median post-hemorrhage BCVA differed significantly between the anti-VEGF (median IQR): 0.79 (0.49–0.82) vs. tPA-gas (median IQR): 1.22 (0.82–1.70) (*P* = 0.008) and anti-VEGF vs. tPA-subretinal-gas -anti-VEGF surgery types (median IQR): 1.85 (1.70-2.00) (*P* = 0.013).

**Fig. 4:**
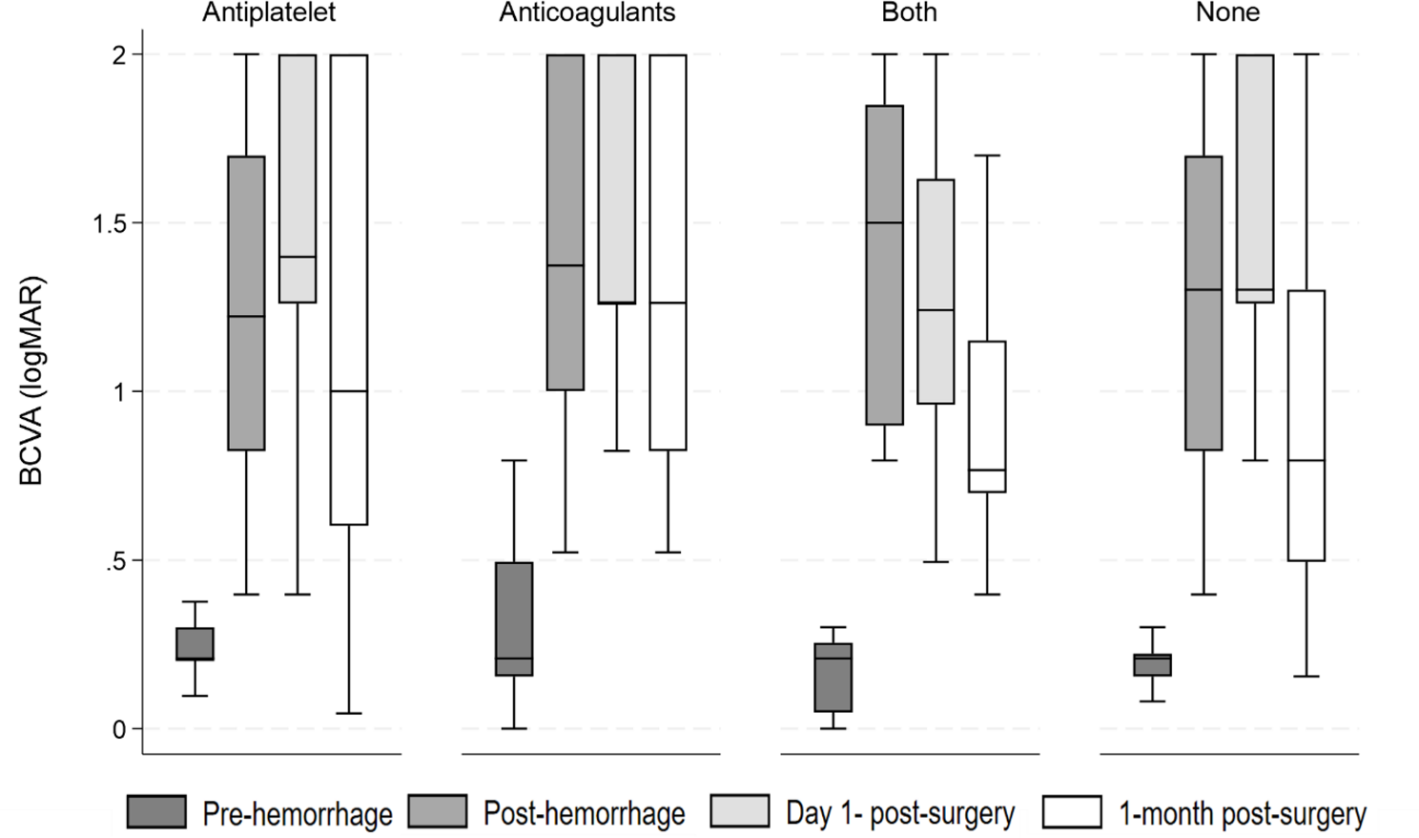
Median BCVA by antiplatelet and/or anticoagulant use. BCVA: Best corrected visual acuity

Figure 4 shows the median BCVA differences at all timepoints among patients using antiplatelets, anticoagulants, a combination of both, or none. The lowest median BCVA (logMAR) at 1-month post-surgery was observed in patients with both and no users, with a median of 0.77 (IQR:0.70–1.15) and 0.80 (IQR: 0.49–1.30), while the BCVA (logMAR) was the highest at post-hemorrhage in the combined group 1.5 (IQR: 0.90–1.85).

BCVA medians differed significantly across all timepoints for antiplatelet users (*P* < 0.05), except between day 1- and 1-month post-surgery (*P* = 1.000). For anticoagulant users, BCVA medians differed significantly between pre- and post-surgery and day 1-post-surgery (*P* < 0.05). For both and none, BCVA medians differed significantly across all timepoints (*P* < 0.05), except between day 1- and 1-month post-surgery (*P* < 0.05).

BCVA medians did not differ between the different antiplatelet and anticoagulant use types.

Supplementary Fig. 1 shows the median BCVA by lens type. There were 58 patients in the phakic - and 62 in the pseudophakic group. The lowest median BCVA (logMAR) at 1-month post-surgery was observed in the phakic group with a median of 0.83 (IQR: 0.49–1.52), while the pseudophakic group had a median of 1.00 (IQR: 0.70-2.00).

BCVA medians differed significantly across all timepoints for both lens types (*P* < 0.001), except between day 1-post-surgery and 1-month post-surgery. BCVA medians did not differ significantly between the phakic and pseudophakic groups at any timepoint.

Supplementary Fig. 2 shows the median BCVA by retinal layers.

Median BCVA differed significantly at all timepoints between the different retinal layers within the groups. The lowest median BCVA (logMAR) could be detected 1 month post-surgery at the combined group with a median of 0.81 (IQR: 0.60–1.30), and the highest median BCVA (logMAR) in both groups post hemorrhage with a median of 1.30 (IQR: 0.82–1.70) and 1.30 (IQR: 0.92–1.70), respectively.

BCVA medians differed significantly across all timepoints for the under neurosensory retina group (*P* < 0.001), except between post-hemorrhage and day 1- (*P* = 0.14) and 1-month post-surgery (*P* = 0.69); and between day 1- and 1-month post-surgery (*P* = 1.000). In the combined group, significant differences were observed in BCVA medians across all timepoints (*P* < 0.05), except between day 1- and 1-month post-surgery (*P* = 1.000). BCVA medians did not differ significantly between the two groups.

## Discussion

SRMH presents a formidable challenge in retinal pathology, leading to severe visual impairment if left untreated. The complexity of SRMH, coupled with its varied etiology, underscores the need for a clear consensus on the most effective treatment strategies. Despite significant advancements in diagnostic and therapeutic options, there remains uncertainty regarding the optimal management approach for SRMH. This retrospective-cohort study analysed 120 cases treated at OUH, aiming to evaluate the effectiveness of five different treatment modalities.

A key observation from our analysis is the impact of treatment timing on visual outcomes. Although there was no statistically significant difference in median BCVA between patients treated within 14 days and those treated later, the median BCVA (logMAR) 1 month post-surgery was higher in the group treated within 14 days (median BCVA 1.00 vs. 0.77). This counterintuitive result suggests that more severe hemorrhages, which prompt quicker intervention, are associated with poorer initial visual acuity. Further, the analysis of patients treated within seven days revealed lower median BCVA (logMAR) 1-month post-surgery compared to those treated later (median BCVA 0.65 vs. 1.00), indicating potential benefits of early intervention, even for initially severe cases. This aligns with findings from Confalonieri, who suggested that very early intervention, particularly within the first 48–72 h of symptom onset, can significantly improve visual outcomes [8].

Additionally, our study highlights the role of hemorrhage size in visual outcomes. Patients with smaller hemorrhages (< 4500 μm) showed lower median BCVA (logMAR) 1 month post-surgery compared to those with larger hemorrhages, though this difference was not statistically significant. This observation supports existing research that smaller hemorrhages, causing less structural disruption, facilitate better visual recovery. The significant difference in post-hemorrhage BCVA between the two groups underscores the impact of larger hemorrhages on immediate visual acuity, corroborated by literature detailing the mechanical and toxic effects of extensive hemorrhages on the retina and RPE [21].

The evaluation of different surgical interventions revealed that the two most utilized treatments, intravitreal tPA + SF6 and intravitreal tPA + SF6 + anti-VEGF, showed significant improvement in BCVA from post-hemorrhage to 1-month post-surgery. This suggests these combinations effectively displace hemorrhage and aid visual recovery, which is consistent with existing research on this topic [22, 23]. However, at the 1-month endpoint no surgical modality demonstrated statistically superior BCVA in comparisons between groups. The only significant differences between groups were observed at the post-hemorrhage timepoint and reflected baseline severity rather than treatment effects.

Beyond our cohort, recent studies inform practice but have not identified a single best procedure. Hillenmayer et al. compared five surgical strategies and observed modest overall visual improvement, with no significant differences between groups. Only one treatment group achieved a statistically significant within-group gain, while more invasive procedures did not yield better postoperative vision and were associated with higher reintervention/complication rates [24]. Maggio et al. reported that intravitreal tPA and gas injection was effective in displacing large SRMH in most eyes and facilitated subsequent management [25]. A recent randomized trial by Murphy et al. suggests that adding tPA to ranibizumab may increase the chance of visual acuity gain in nAMD-related SRMH, and the authors recommend further clinical trials [26]. To address remaining uncertainty, Lee et al. published an updated protocol for the pan-European TIGER randomized trial, comparing pars plana vitrectomy with subretinal tPA and gas versus intravitreal tPA and gas. However, trial results have not yet been published [27]. Consistent with this broader pattern, He and colleagues did not find intravitreal combinations to consistently outperform subretinal tPA, and a systematic review by Confalonieri likewise found no clear superiority across modalities [28, 29]. Given the variability in patient populations and procedures, and the small size of several subgroups, determining superiority is difficult and highlights the need for larger prospective trials with standardized protocols and longer follow-up to establish the most effective approach.

At the patient level, visual outcomes can vary with lens status. In our cohort, phakic eyes showed better median 1-month BCVA than pseudophakic eyes (0.83 vs. 1.00 logMAR), however, the subgroups were too small to show statistical significance.The difference in visual outcomes might be related to lower levels of Pigment Epithelium-Derived Factor (PEDF) in pseudophakic eyes, which have been associated with improved retinal health and function [30].

The use of antiplatelet and anticoagulant medications was not associated with significant between-group differences in BCVA at any timepoint. At 1 month, the lowest median BCVA (logMAR) was observed in the “both” and “none” groups, whereas the antiplatelet-only and anticoagulant-only groups had higher medians.

The involvement of different retinal layers impacted visual outcomes, though the differences in BCVA 1-month post-surgery were not statistically significant. Patients with combined involvement had the lowest median BCVA (logMAR) 1-month post-surgery. The highest median BCVA (logMAR) was observed in patients with hemorrhage under the neurosensory retina. This pattern suggests that the location of hemorrhage relative to retinal layers may influence photoreceptor disruption and visual recovery, as hemorrhage at the subretinal level may be more harmful to photoreceptors.

However, it is important to acknowledge the limitations of this study when interpreting these results. A key limitation of this study is the uneven distribution of patients across treatment groups, with some subgroups being underpowered (e.g., *N* = 4). While the overall cohort had sufficient power to detect clinically relevant differences in treatment timing and hemorrhage size, subgroup analyses, particularly for surgical modalities, lacked statistical power due to small sample sizes. Being a retrospective cohort study, it does not establish causal relationships between BCVA outcomes and exposure variables, but this was not the primary focus. Nonetheless, this study provides a useful foundation for planning future advanced research, which could explore these relationships further.

## Conclusions

In conclusion, our study underscores the complexity of SRMH management and the importance of individualized treatment strategies. In this cohort, starting treatment within 7 days was associated with better 1-month BCVA, while a 14-day cutoff showed no benefit. Combination therapies with tPA and SF6 gas, with or without anti-VEGF, showed significant within-group improvement at 1 month, but no modality was superior at that endpoint. Further prospective studies are needed to establish definitive guidelines and refine treatment approaches. The findings suggest that the timing and type of intervention should be carefully personalized to each patient’s specific circumstances to maximize visual recovery.

## Supplementary Information

Below is the link to the electronic supplementary material.

Supplementary Material 1

## Data Availability

Data is provided within the manuscript or upon request from the corresponding author.

## Abbreviations

SRMH: Subretinal Macular Hemorrhage
RPE: Retinal Pigment Epithelium
BCVA: Best-Corrected Visual Acuity
tPA: Tissue Plasminogen Activator
SF6: Sulfur Hexafluoride
VEGF: Vascular Endothelial Growth Factor
anti-VEGF: Anti-Vascular Endothelial Growth Factor
OCT: Optical Coherence Tomography
OUH: Oslo University Hospital
IQR: Interquartile Range
nAMD: Neovascular Age-Related Macular Degeneration
Q-Q: Quantile-Quantile
PEDF: Pigment Epithelium-Derived Factor
