# Supplementary material for: Outcomes of subretinal macular hemorrhage treatment: a 7-year retrospective cohort study at Oslo University Hospital
Source: Int J Retina Vitreous. 2025 Nov 22;11:143. doi: 10.1186/s40942-025-00749-3 (PMC12751555; doi:10.1186/s40942-025-00749-3)
Supplement: Supplementary file 1 — Supplementary Material 1 [file 40942_2025_749_MOESM1_ESM.docx]

**Supplementary Figures**

**Supplementary Figure 1:** Median BCVA by lens type

**Supplementary Figure 2:** Median BCVA by retinal layers
